# Radiotherapy versus low-dose tamoxifen following breast-conserving surgery for low-risk and estrogen receptor-positive breast ductal carcinoma in situ: an international open-label randomized non-inferiority trial (TBCC-ARO DCIS Trial)

**DOI:** 10.1186/s12885-023-11291-6

**Published:** 2023-09-14

**Authors:** Sung-Hsin Kuo, Ling-Ming Tseng, Shou-Tung Chen, Yasuaki Sagara, Yuan-Ching Chang, Hsien-Tang Yeh, Yao-Lung Kuo, Chih-Chiang Hung, Tzu-Pin Lu, Yi-Hsuan Lee, Masakazu Toi, Chiun-Sheng Huang

**Affiliations:** 1 Department of Oncology, National Taiwan University Hospital, Taipei, Taiwan; 2 Graduate Institute of Oncology, National Taiwan University College of Medicine, Taipei, Taiwan; 3 Department of Radiation Oncology, National Taiwan University Cancer Center, National Taiwan University College of Medicine, Taipei, Taiwan; 4 Cancer Research Center, National Taiwan University College of Medicine, Taipei, Taiwan; 5 Department of Surgery, Taipei Veterans General Hospital, Taipei, Taiwan; 6 Department of Surgery, Changhua Christian Hospital, Changhua, Taiwan; 7 Department of Breast Surgical Oncology, Hakuaikai Social Cooperation, Sagara Hospital, Kagoshima, Japan; 8 Department of Surgery, Mackay Memorial Hospital, Taipei, Taiwan; 9 Department of Surgery, Lotung Poh-Ai Hospital, Yilan, Taiwan; 10 Division of Breast Surgery, Department of Surgery, National Cheng Kung University Hospital, Tainan, Taiwan; 11 Department of Surgery, Division of Breast Surgery, Taichung Veterans General Hospital, Taichung, Taiwan; 12 Department of Public Health, National, Institute of Epidemiology and Preventive Medicine, Taiwan University, Taipei, Taiwan; 13 Department of Pathology, National Taiwan University Hospital and National Taiwan University, College of Medicine, Taipei, Taiwan; 14 Tokyo Metropolitan Cancer and Infectious Disease Centre, Komagome Hospital, Tokyo, Japan; 15 Department of Surgery, National Taiwan University Hospital and National Taiwan University College of Medicine, No. 7, Chung-Shan South Rd, Taipei, Taiwan

**Keywords:** Ductal carcinoma in situ, Breast, Tamoxifen, Radiotherapy, Low-risk

## Abstract

**Background:**

Radiotherapy (RT) following breast-conserving surgery (BCS) is mainly used to decrease the rate of ipsilateral breast tumor recurrence (IBTR) in women with breast ductal carcinoma in situ (DCIS). Recent studies have demonstrated that low-dose tamoxifen significantly reduces IBTR in breast DCIS. Here, we aim to determine whether the administration of low-dose tamoxifen is non-inferior to RT in preventing IBTR in patients with low-risk characteristics of breast DCIS.

**Methods/design:**

This is a prospective, international, open-label, randomized, non-inferiority trial. Patients with low-risk clinicopathologic features (> 40 years old, low risk of breast cancer susceptibility gene (*BRCA) 1* and *BRCA2* mutations, mammographically detected unicentric and non-mass lesions, low- or intermediate-grade without comedo or necrosis, measuring < 2.5 cm with margins ≥ 3 mm, and estrogen receptor-positive status) of DCIS who underwent BCS will be randomized at a 1:1 ratio to either receive tamoxifen (5 mg/day) for 5 years or undergo RT with conventional fractions (50 Gy in 25 fractions) or hypofractionations (40.05 Gy in 15 fractions). Randomization will be stratified by the Taiwan Breast Cancer Consortium. As approximately 5% of patients cannot tolerate the side effects of low-dose tamoxifen and will receive RT, we estimate that 405 patients will be randomized to a low-dose tamoxifen arm and 405 patients to the RT arm, according to a non-inferiority margin within 5% of IBTR difference and 90% β-power noticing non-inferiority. The primary endpoints are breast tumor recurrence, including ipsilateral, regional, contralateral, and distant recurrence of breast DCIS or invasive cancer. The secondary endpoints are overall survival and adverse effects of RT and tamoxifen. Translational studies will also be conducted for this trial.

**Discussion:**

This is the first non-inferiority trial on breast DCIS. This study will provide an important recommendation for clinical physicians on whether to use low-dose adjuvant tamoxifen for patients with low-risk breast DCIS who do not want to receive adjuvant RT.

**Trial registration:**

ClinicalTrials.gov, ID: NCT04046159, Registered on April 30, 2019.

**Supplementary Information:**

The online version contains supplementary material available at 10.1186/s12885-023-11291-6.

## Background

### Background and rationales

#### Low-risk characteristics of breast ductal carcinoma in situ (DCIS)

Radiotherapy (RT) following breast-conserving surgery (BCS) is commonly used to decrease local recurrence of breast ductal carcinoma in situ (DCIS) [1]. However, previous retrospective studies suggest that certain patients with breast DCIS who had low-risk characteristics and underwent BCS alone do not develop a recurrent invasive breast cancer or DCIS over time [2]. To identify a substantial proportion of breast DCIS patients with low-risk characteristics who can be spared adjuvant RT, two prospective trials, including the Eastern Cooperative Oncology Group (ECOG) E5194 and one trial designed by Dana Farber/Harvard Cancer Institute, and one randomized trial (Radiation Therapy Oncology Group [RTOG] 9804), enrolled breast DCIS patients with low-risk characteristics, including lower histological grade, smaller size, and larger margin width, and reported the ipsilateral breast tumor recurrence (IBTR) rate in this subgroup of patients [3–6].

In the Dana Farber/Harvard Cancer Institute trial, Wong et al. showed that the 5-year IBTR rate was 12% in breast DCIS patients who met the criteria of low- to intermediate-grade disease, had a margin width of 10 mm, and had undergone BCS alone [3]. In the E5194 trial, 7-year and the 12-year IBTR rates of 10.5% and 14.4%, respectively, were observed in patients with low-risk cohort-1 breast DCIS (cohort 1 criteria: mammographically detected low- or intermediate-grade DCIS, measuring < 2.5 cm with surgical margins ≥ 3 mm) who underwent BCS alone [4, 5]. Notably, the risk for developing DCIS and invasive cancer in low-risk cohort-1 breast DCIS of the ECOG trial increased gradually over time without plateau [4, 5]. In a randomized trial of RTOG 9804 comparing the occurrence of IBTR among patients who underwent RT and were observed for breast DCIS with similar tumor characteristics in cohort 1 of the ECOG E5194 trial, McCormick et al. showed that IBTR rates were higher in those without RT than in those with RT (7-year IBTR rate, 6.7% vs. 0.9%, *P* < 0.001) [6]. In our cohort of 150 breast DCIS patients who underwent BCS alone and whose clinicopathological features met the criteria of cohort 1 of the ECOG E5194 trial, we found a 7-year IBTR rate of 5.6% [7]. Based on the data from the ECOG E5194 and RTOG 9804 trials and our cohort study, the 7-year IBTR rate ranged from 5.6%–10.5% in breast DCIS patients who underwent BCS alone and had low-risk characteristics [4–7], indicating that RT following BCS is still desirable for patients who had pathological features similar to ECOG E5194 cohort 1.

The randomized trial UK/ANZ (UK, Australia, and New Zealand) DCIS trial, which enrolled breast DCIS patients irrespective of low-risk or high-risk characteristics, showed that among patients who underwent BCS alone, administration of tamoxifen (20 mg/day for 5 years) significantly reduced ipsilateral (Hazard ratio [HR], 0.77; 95% confidence interval [CI], 0.59–0.98; *P* = 0.04) and contralateral breast events (HR, 0.27; 95% CI, 0.12–0.59; *P* = 0.001). However, the benefit of tamoxifen in reducing ipsilateral breast events (HR, 0.93; 95% CI, 0.50–1.75; *P* = 0.8) was not observed in the BCS plus RT group [8]. These findings suggest that the addition of tamoxifen after RT may not reduce IBTR in breast DCIS patients who had low-risk characteristics, like in cohort 1 of the ECOG E5194 trial. However, the association between IBTR and estrogen receptor (ER) status in tumors of patients with DCIS who entered the aforementioned trials was not assessed [4–6, 8].

Among 732 patients (41%) with breast DCIS who received tamoxifen from the original National Surgical Adjuvant Breast and Bowel Project B-24 population, in which ER-positive tumors were 76%, Allred et al. showed that tamoxifen administration significantly diminished ipsilateral and contralateral recurrent breast events in those with ER-positive tumors, but did not impede recurrent events in those with ER-negative tumors [9]. In addition, we observed that among breast DCIS patients who underwent BCS alone, age < 40 years and negative ER status in tumors were two prognostic factors for increasing IBTR [7]. Our study also revealed that, even among breast DCIS patients who had pathological features similar to those in cohort 1 of the ECOG E519 trial, ER-negative status was associated with a trend of higher 7-year IBTR (8% vs. 5%, *P* = 0.69) [7].

#### Rationale and hypotheses underlying this trial

Although the results obtained from cohort 1 of the ECOG E5194 trial and the no RT group of the RTOG 9804 trial showed a 7-year IBTR rate ranging from 5.6%–10.5 [4–6], both trials enrolled a proportional number of younger patients and those with ER-negative tumors. Several studies, including our own, showed that age < 40 years and ER-negative tumor were associated with higher IBTR in patients with breast DCIS [7, 10–12]. In addition, the UK/ANZ randomized trial demonstrated the significant benefit of tamoxifen administration in lessening IBTR in breast DCIS patients undergoing BCS alone, but the aforementioned impacts were not observed in those undergoing BCS followed by RT [8]. In a randomized trial (TAM-01) comparing low-dose tamoxifen (5 mg/day for 3 years) with observation alone for preventing local recurrence in women who had hormone receptor-positive (ER or progesterone receptor ≥ 1%) intraepithelial neoplasia (including atypical ductal hyperplasia and lobular or ductal carcinoma in situ) after BCS, DeCensi et al. showed a significant decrease in ipsilateral and contralateral recurrent events in patients who received low-dose tamoxifen than those without, in which RT was preserved for patients with high-risk factors of DCIS [13]. This randomized trial also reported rare serious adverse events of low-dose tamoxifen (12/249 [4.8%]); only one patient experienced deep vein thrombosis and another developed stage I endometrial cancer [13]. However, the influence of low-dose tamoxifen in preventing IBTR is similar to that of the RT in breast DCIS patients whose pathological tumor features are similar to the criteria of ECOG E5194 cohort 1 and have a positive ER status, but its effects remains uncertain.

For patients with breast DCIS aged > 40 years and with pathological tumor features meeting the ECOG E5194 cohort 1 criteria and positive ER, we hypothesized that the administration of low-dose tamoxifen is not inferior to the prescription of RT in terms of reducing the IBTR.

### Objectives

The aim of this study is to explore whether the clinical efficacy of administration of tamoxifen (5 mg/day) for 5 years following BCS is not inferior in reducing recurrence of breast events when compared with RT following BCS in breast DCIS patients with low-risk clinicopathologic features and positive ER status in tumors.

### Trial design

**Fig. 1:**
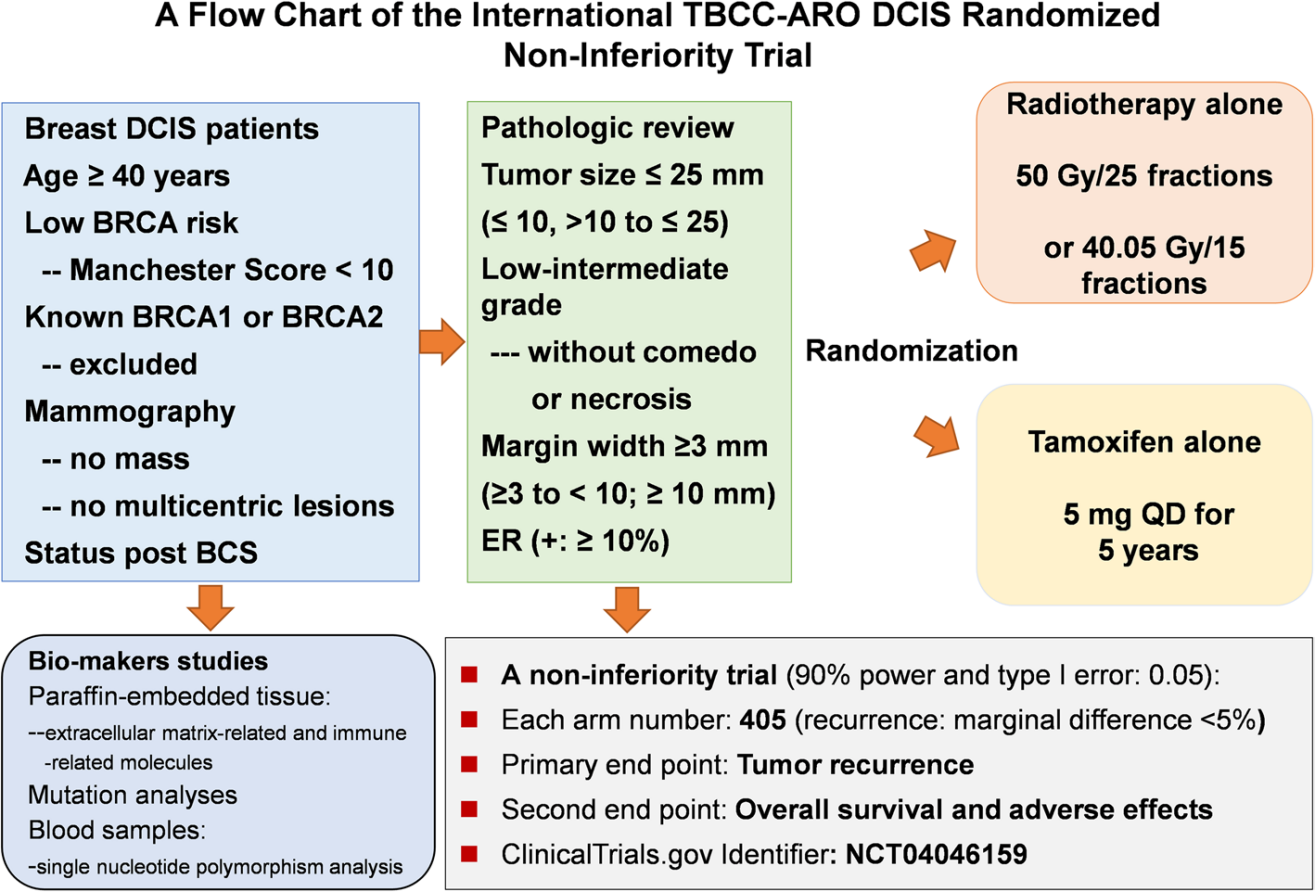
Schematic diagram of the design of an international open-label randomized non-inferiority trial of whole breast radiotherapy versus low-dose tamoxifen for low-risk and estrogen receptor-positive breast ductal carcinoma in situ after breast conserving surgery. Abbreviations: DCIS, ductal carcinoma in situ; BCS, breast conserving surgery; SNP, single nucleotide polymorphism

The study outline of the Taiwan Breast Cancer Consortium (TBCC)- Japan Academic Research Organization (ARO) is a prospective, international, open-label, randomized, non-inferiority trial. A flowchart of the TBCC-ARO DCIS trial is shown in Fig. 1. Patients aged > 40 years with low-risk characteristics of breast DCIS and who have undergone BCS may be enrolled in this study and will be randomly assigned to either the RT or low-dose tamoxifen groups for 5 years.

### Methods: participants, interventions, and outcomes

#### Study setting

This international trial consists of 11 participating medical centers: 9 sites from the TBCC and 2 sites from the ARO.

### Eligibility criteria

#### Inclusion criteria

1. New histologically diagnosed breast DCIS
2. Age ≥40 years
3. Low risks of *BRCA1* and *BRCA2*: Manchester Score < 10 (Supplementary Table 1) [14, 15].
4. DCIS must be detected by a mammogram and must be unicentric, with non-mass lesions
5. Status post-BCS
6. Pathological characteristics:
  6.1 Lesions ≤2.5 cm in the greatest dimension on pathology specimens (use the largest measured size from the pathology report to obtain the required measurement of ≤2.5 cm)
  6.2 Must be classified as low or intermediate nuclear grade DCIS but without comedo or necrosis, according to the Pathologic Guidelines [16, 17].
  6.3 Margins, as assessed by the ink method, should be ≥ 3 mm.
  6.4 Must be ER-positive DCIS, and the ER percentage must be ≥ 10%
7. Clinically node-negative

#### Exclusion criteria

1. Known *BRCA1* or *BRCA2* mutations
2. Women whose DCIS is palpable at the time of diagnosis, multicentric (mammography), mass (mammography), or bloody nipple discharge
3. Pathological characteristics:
  3.1 Lesions measuring >2.5 cm in the greatest dimension on pathology specimens
  3.2 High-grade lesions or low- to intermediate-grade with comedo or necrosis classified according to the Pathologic Guidelines [16, 17].
  3.3 Margins, as assessed by the ink method, were < 3 mm.
  3.4 ER-negative DCIS or ER-positive percentage < 10% in tumor cells
4. Post-mastectomy patients.
5. Prior diagnosis of neoplasm within 5 years, except for cervical intraepithelial neoplasia 1 or localized non-melanomatous skin cancer
6. Evidence of clinically significant cardiac disease, as defined by cardiac disease (New York Cardiac Disease Grade II), history of myocardial infarction, cerebral stroke, unstable arrhythmia, and unstable angina pectoris within 12 months before study entry

### Interventions

#### Intervention description

In the current study, patients who are randomized to the RT arm will undergo RT with 50 Gy divided in 25 fractions (2 Gy each) or with 40.05 Gy divided in 15 fractions (2.67 Gy each); RT dose and fractions will be dependent on the institution’s experience. RT for treating the whole breast will be delivered daily from Monday to Friday; however, regional lymphatic irradiation and boost of the tumor bed will not be allowed.

All patients randomized to the tamoxifen treatment arm will receive tamoxifen (5 mg/day) for 5 years. Tamoxifen toxicities, including hot flashes, transient nausea, and vaginal discharge, are minimal, whereas skin rash, vaginal bleeding, edema, and embolisms seldom occur. As the drug compliance of patients who are randomized to low-dose tamoxifen is crucial, the adherence to taking 5 mg tamoxifen was defined as the use of at least 90% of these pills each year for at least 5 years.

### Criteria for discontinuing or modifying allocated interventions

If the inclusion and/or exclusion criteria are contravened after recruitment, these patients will be removed from the trial. Dropouts from this trial will receive adjuvant RT with or without 20 mg tamoxifen based on clinicopathological features. For these patients, their clinical data and treatment characteristics will be retained, unless they unambiguously refuse.

### Strategies to improve adherence to interventions

Participants will follow the recommendations of the study protocol, including the doses of RT and tamoxifen. Because the study is divided into two arms, adjuvant RT and adjuvant low-dose tamoxifen, deviations in the RT or tamoxifen doses will not be allowed. If participants refuse the assigned treatment, they will remain in the trial’s intention-to-treat analysis, unless they unambiguously refuse.

### Relevant concomitant care permitted or prohibited during the trial

In this study, we will allow participants who take medication necessary for the performance of either examination or procedure and take previously prescribed medications before randomization.

### Provisions for post-trial care

For participants in Taiwan, treatments, including administration of tamoxifen and RT, and post-treatment follow-ups are covered by public health insurance.

### Outcomes

Our primary objective is to observe breast tumor recurrence, including ipsilateral, regional, contralateral, and distant recurrences of breast cancer (DCIS or invasive cancer events).

Our secondary objective is to assess overall survival in all patients, adverse effects of RT and tamoxifen, and translational studies—including single nucleotide polymorphisms (SNPs), extracellular matrix (ECM)- and immune-related molecules, and mutational analyses of selected genes.

### Translational studies

Pathologically, tumor cells of DCIS are surrounded by a dynamic layer of ECM, including myoepithelial cells (MECs), basement membranes, immune cells, fibroblasts, and stroma [18]. These molecules within the microenvironments of DCIS may participate in the progression of DCIS to invasive ductal carcinoma (IDC) or enhance DCIS cells to migrate, thus causing recurrence and metastases [18]. MECs can upregulate the expression of tumor suppressor proteins, such as maspin, avβ6, MMP8, and TIMP-1, and may drive progression of DCIS to IDC tumor cells [19, 20].　We also showed that stromal CD10 (a cell surface zinc-dependent metalloproteinase [21]) expression (*P* = 0.024) and loss of a tumor suppressor gene retinoblastoma (RB) [22] expression (*P* = 0.01) were closely associated with a higher incidence of IBTR in breast DCIS through multivariate analyses [23].

Regarding studies on the role of immune cells in the biology of breast DCIS [24], Hendry et al. reported that tumor infiltrating lymphocytes (TILs) were significantly greater in high-grade, ER-negative, and HER2-positive tumors of DCIS [25]. Furthermore, the TIL score was significantly higher in cases with *TP53* mutations [25]. The authors also found that the positive rate for programmed death-ligand 1 (PD-L1) was rarely present in tumor parts (11%) when compared with immune cell parts (67%) [25]. Interestingly, PD-L1 expression found in any part of tumor or immune cells was significantly associated with ER-negative and HER2-positive status in breast DCIS [25]. However, Thompson et al. showed that none of the DCIS cells expressed PD-L1, but most of the DCIS-surrounding infiltrating lymphocytes expressed PD-L1 [26].

To evaluate the association between breast cancer susceptibility loci and the risk of DCIS, Campa et al. genotyped 39 SNPs correlated with the risk of invasive breast cancers in three groups, including 1,317 cases of breast DCIS, 10,645 cases of invasive breast cancer, and 14,006 healthy controls [27]. They showed that five SNPs of *CDKN2BAS*_rs1011970, *FGFR2*_rs3750817, *FGFR2*_rs2981582, *TNRC9*_rs3803662, and 5p12_rs10941679 were closely correlated with the development of breast DCIS (*P* < 0.0016) [27]. Previously, we reported that *MAP3K1*_rs889312 (C/C) significantly correlated with a worse prognosis in patients with early-stage breast cancer who received adjuvant hormonal therapy alone (no chemotherapy) [28]. In addition, mutated *MAP3K1* was found in tumor cells of synchronous DCIS with IDC [29, 30]. In breast DCIS cases, we found that *MAP3K1* expression was closely associated with a higher 7-year IBTR rate (36.8% vs. 14.3%, *P* = 0.015) [23]. Furthermore, we showed that inhibition of *MAP3K1* significantly reduced cell growth and migration and NF-κB-dependent genes; it also enhanced the cytotoxicity of tamoxifen in two ER-positive IDC cell lines (MCF-7 and T-47D*,* to mimic DCIS) [31].

Based on the aforementioned results, we will explore which biomarkers are associated with local recurrence of DCIS. The translation studies will be divided into three parts: (1) SNPs analysis (DNA extracted from mononuclear cells obtained from peripheral blood)—including *CDKN2BAS*_rs1011970, *FGFR2*_rs3750817, *FGFR2*_rs2981582, *TNRC9*_rs3803662, 5p12_rs10941679, *CYP2B6*_rs3211371, and *MAP3K1*_rs889312—will be performed in all patients who agree to participate in the translational study; (2) the expression pattern of ECM-related molecules—including CD10, MAP3K1, retinoblastoma, maspin, αvβ6, MMP-8, MMP-9, TIMP-1, TGF-β—and immune cell-related molecules—including CD4, CD8, regulatory T cells (FoxP3 +), PD-L1, macrophages, and myeloid-derived suppressor cells *(*MDSC)—will be assessed by immunohistochemical staining in recurrent cases and in selected matched non-recurrent controls; and (3) mutations in genes such as *TP53, PIK3CA, GATA3, MLL3, and MAP3K1* in DNA extracted from paraffin-embedded tissue specimens will be analyzed to understand the relationship between the expression of these genes and clinical outcomes in patients.

### Participant timeline

The participant timeline is presented in Table 1. Clinical assessments, including physical examinations and adverse events resulting from RT or low-dose tamoxifen, will be recorded in a case report form. During the follow-up period, participants will undergo breast ultrasound, mammography, and chest radiography annually.

**Table 1:** Participant timeline

	**Study Period of Non-inferiority trial**							
**Characteristics**	Enrollment	Allocation	Post Allocation					
Time point	-6 weeks	0	0–3 months	6 months	9 months	12 months	Within 2–5 years	5–10 years
Enrollment:								
Eligibility screen	V							
Informed consent	V							
Randomization	V							
Allocation		V						
Interventions:								
Radiotherapy			V					
Tamoxifen			V					
Assessments:								
Physical examination	V		V	V	V	V	Every 3 months	Every 6 months
KPS	V		V	V	V	V	Every 3 months	Every 6 months
Complete blood count	V		V		V		Every 6 months	Annually
Serum biochemistry	V		V		V		Every 6 months	Annually
Adverse events			V	V	V	V	Every 3 months	Every 6 months
Breast ultrasound^a^				V			Annually	Annually
Mammography^a^					V		Annually	Annually
Chest X-ray						V	Annually	Annually

*Abbreviation*: *KPS* Karnofsky performance score

^a^based on the BIRADS score, if BIRADS 3, suggestion, follow-up every 6 months

### Sample size

This study is designed to evaluate whether the efficacy of low-dose tamoxifen in reducing IBTR is not inferior to that of whole breast irradiation in patients with low-risk and ER-positive breast DCIS. We hypothesize that in all patients who meet the cohort 1 criteria of the ECOG E5194 study and have ER-positive status in the tumor, the 7-year IBTR rate in those who underwent BCS alone will be < 10.5% (assumption: 6–7%). In our previous study, breast DCIS patients who underwent BCS alone had the ECOG E5194 cohort 1 criteria, in whom the 7-year IBTR rate was 5% and 8% in the ER-positive and ER-negative groups, respectively [7]. For the ER-positive group tumors, most patients received tamoxifen as an adjuvant setting. Under these assumptions, we hypothesized that for low-risk breast DCIS, the 7-year IBTR rate would be approximately 6% in patients who underwent BCS alone (neither tamoxifen nor RT). The current trial was designed to evaluate whether the administration of low-dose tamoxifen (tamoxifen 5 mg for 5 years; experimental group) is not inferior in decreasing the cumulative incidence of IBTR when compared with the prescription of RT (50 Gy in 25 fractions or 40.05 Gy in 15 fractions; control group). We assume that a 6% incidence of IBTR at 7 years will be observed in participants who underwent whole breast RT alone and expect that the cumulative incidence of 7-year IBTR will not be > 6% in participants who receive low-dose tamoxifen (5 mg for 5 years).

In total, 385 patients are needed in each group to provide 90% β-power with an α type I error of 5% (margin, IBTR difference, 5%); this permits a loss of 5% of patients due to follow-up at 5 years (statistical methods are shown in Table 2). As approximately 5% of patients cannot tolerate the side effects of low-dose tamoxifen and will receive RT, we estimate that 405 patients will be enrolled in the tamoxifen arm. Therefore, 810 patients will be enrolled in this randomized non-inferiority trial. The targeted number of breast events is not described in the protocol, but the ripeness of the data from this trial will be revaluated and deliberated by Taiwanese and Japanese teams. If at least 80% of case report forms are returned 5 years after starting treatment, we will properly consolidate this trial’s corrected data.

**Table 2:** Statistical methods

**α: type 1 error—> 0.05**
**β: power—> 0.9**
**Assume (no-RT) low risk IBTR: 6%**
**Assume that Tamoxifen = RT = 6%**
**Assume a margin (IBTR difference: 5%)**
**N = (Z**_**α**_** + Z**_**β**_**)**^**2**^**xS**^**2**^**/Δ**^**2**^ = **385 (per arm)**
N = 2 * n = 770 (total)

### Recruitment

Patients will be recruited among those with breast DCIS who underwent BCS and visited the outpatient department of participating sites of the TBCC-ARO DCIS trial. In the current protocol, no advertising or inducements will be permitted and recruited. Patients will be randomized by the TBCC-developing “Clinical Study Information System” (CSIS) by logging clinicopathological information on their respective websites.

### Assignment of interventions: allocation

#### Sequence generation

When patients meet the criteria of the current study, they will be stratified into different groups based on the pathological characteristics of their post-BCS tumor specimens (tumor size: ≤ 1 cm vs. > 1 cm; margin width: 3– < 10 mm vs. ≥ 10 mm) before randomization.

#### Concealment mechanism

In this study, randomization of patients with DCIS will be performed using the randomization module found in the CSIS website of the TBCC-ARO DCIS trial. The TBCC-ARO DCIS central site will be allocated a concealment of randomization.

#### Implementation

In this study, information on adjuvant RT and low-dose tamoxifen following BCS after randomization will be provided to participants who meet the inclusion criteria at the outpatient clinic. When written informed consent is obtained from participants, the participating investigators will introduce information on patients’ clinicopathological characteristics to the CSIS website of the TBCC-ARO trial via a secured module. Furthermore, each patient will receive a code number, and they will be randomized to the RT or low-dose tamoxifen arms after successful registration.

### Assignment of interventions: determining who will be blinded

The current protocol of the TBCC-DCIS trial is an international, open-label, randomized, non-inferiority trial. Therefore, no blinding is planned.

### Procedure for unblinding if needed

Because this is an open-label, randomized, non-inferiority trial, methods for blinding are not indicated.

### Data collection and management

#### Plans for assessment and collection of outcomes

After randomization, all patients will undergo annual mammography, hemogram, and biochemistry examinations every 6 months or every 12 months, for a total of 10 years (Table 1). The toxicity of RT will be assessed during RT or after its completion, while the toxicity of tamoxifen will be assessed after completion of its administration. Ipsilateral recurrence, contralateral recurrence, and survival will be assessed after entering the clinical trial.

### Plans to promote participant retention and complete follow-up

#### Data management

When the current trial is ongoing, its data will be accessed by the Data Safety and Monitoring Committee (SMC) and Statisticians of TBCC-ARO. The principal investigator has full responsibility for the assessment of clinical data, which will be available for the principal investigator and co-investigators, if the clinical trial is finished. Clinical data, including name, contract information, subject number, informed consent, pertinent medical history, result of key parameters of trials, adverse events, recurrence, and clinical outcomes of participants, will be sent to the CSIS website of the TBCC-ARO trial, and these data and documents will be stored for 10 years after trial completion or according to the regulatory requirements.

### Statistics

#### Statistical methods for primary and secondary outcomes

In this study, patients who do not follow the protocol of their assigned treatment arm will not be analyzed. We will use the chi-square test, Student’s *t*-test, and Fisher’s exact test to compare the clinical characteristics between patients undergoing RT and those receiving low-dose tamoxifen. In this study, we will use the Kaplan–Meier method to estimate the time to IBTR (only breast area), ipsilateral regional recurrence of lymph nodes (including axillary, supraclavicular, or internal mammary lymph nodes), contralateral breast local recurrence, and distant metastases from the date of surgical excision. In addition, we will use this method to calculate overall survival, which will be measured from the date of surgical excision to the date of death from any cause or to the date of the last follow-up. Comparisons between the patients will be performed using the log-rank test. All statistical analyses will be carried out at a 5% significance level.

### Methods for additional analyses

In this trial, the subgroups of pathological features will be managed based on the primary and secondary endpoints stratified by tumor size and surgical margin status.

### Interim analyses

We will perform interim analyses if the 5-year IBTR is > 5% in patients who received only low-dose tamoxifen or only underwent RT; furthermore, we will discontinue this non-inferiority trial according to the assumption that the cumulative incidence of IBTR in the experimental (tamoxifen) or control groups (RT) was 6% at 7 years.

### Adverse event reporting and harms

The assessment of acute toxicities (≤ 90 days from the start of RT) and adverse effects of tamoxifen will be scored using the Common Terminology Criteria for Adverse Events (CTCAE), version 4.03 [32]. We will score late RT toxicities (> 90 days from the start of treatment) using the RTOG/European Organization for Research and Treatment of Cancer Late Radiation Morbidity Scheme [33].

We will record serious adverse events (SAEs), which must fulfill at least one of the following criteria: (1) an unexpected event that immediately results in risk of death; (2) the events require patients to be hospitalized for further care or prolong hospitalization; and (3) the events cause significant or persistent incapacity or disability.

## Discussion

We previously demonstrated that among patients who did not receive RT in our institution (National Taiwan University Hospital [NTUH] cohort), ER-negative status was significantly correlated with a higher IBTR rate [7]. We also collected the clinicopathological features of 1,964 patients from 12 Taiwan hospitals (Taiwan cohort) and assessed the relationship between ER status and IBTR in breast DCIS patients who underwent BCS [34]. We found that in patients who did not receive RT, those with ER-negative DCIS had a higher 7-year IBTR than those with ER-positive DCIS (23.9% vs. 5.4%, *P* = 0.007) [34]. The results of the NTUH and Taiwan cohorts demonstrated that among patients with breast DCIS who underwent BCS alone, ER-negative status in tumors was closely associated with a higher IBTR. Our results are consistent with previous reports showing that patients with ER-negative tumors had a higher incidence of local recurrence than those with ER-positive tumors after undergoing BCS without RT [10, 35, 36].

In addition to ER-negative status in tumors, we previously reported that age < 40 years was significantly associated with the risk of IBTR in breast DCIS patients who underwent BCS alone [7]. Cronin et al. also demonstrated that age < 40 years was a prognostic factor for local recurrence in patients with breast DCIS who underwent BCS with and without RT [11]. Tunan-de-Lara et al. also reported that age ≤ 40 years was independently associated with higher local recurrence of breast DCIS regardless of the pathological features of the tumor [12].

For patients with low-risk DCIS who underwent BCS alone in the ECOG E5194 and RTOG 9804 trials [4–6], the risk for DCIS and invasive cancer increased slightly, indicating that RT is needed for this subtype of breast DCIS. However, a proportion of patients who were aged < 40 years and those who had ER-negative tumors—who were at risk of IBTR if they underwent BCS without RT—were enrolled [4–6]. Because tamoxifen has demonstrated higher clinical efficacy in reducing local recurrence in ER-positive than in ER-negative DCIS [9], it is reasonable to hypothesize that the efficacy of low-dose tamoxifen in decreasing local recurrence is not inferior to RT in patients aged > 40 years with low-risk (cohort 1 characteristics of the ECOG E5194 trial) and ER-positive breast DCIS.

Nevertheless, tamoxifen has an estrogenic effect on the endometrium and has been reported to increase the risk of endometrial cancer; tamoxifen-associated endometrial cancers are a localized disease with rare morbidity [37, 38]. Previous studies also demonstrated that the administration of low-dose tamoxifen (1–5 mg/day) had biological effects on breast cancer in terms of the Ki-67 index and lower endometrial cell proliferation [39, 40]. In a single institute experience with tamoxifen in the adjuvant setting for patients with breast DCIS, Guerrieri-Gonzaga et al. found that low-dose tamoxifen (5 mg per day) reduced ipsilateral recurrence in women aged > 50 years with ER-positive DCIS [41]. In a median follow-up of 9.7 years of a randomized trial of TAM-01, the 3-year administration of low-dose tamoxifen (5 mg for 3 years) resulted in a significant decline of 50% recurrence in the patients with DCIS without increased adverse effects [42]. Considering that low-dose tamoxifen significantly decreased local recurrence and caused fewer side effects in patients with breast DCIS from the data of a randomized trial (TAM-01) [13, 42], we theorized that low-dose tamoxifen is similar to standard-dose tamoxifen in terms of preventing recurrence in low-risk DCIS patients.

In conclusion, this prospective study aims to evaluate whether low-dose tamoxifen administration is not inferior to RT in decreasing local recurrence in women aged > 40 years with low-risk characteristics and ER-positive breast DCIS who have undergone BCS. If this study confirms its primary endpoint, it will provide an important alternative for clinical physicians to recommend adjuvant low-dose tamoxifen for patients with low-risk breast DCIS who do not want to receive adjuvant RT because of potential side effects. Moreover, the cost of low-dose tamoxifen is lower than that of RT. We believe that this non-inferior trial will be helpful in prospective clinical practice in patients with specific low-risk breast DCIS.

### Trial status

The Research Ethics Committee C of the National Taiwan University Hospital approved the protocol version 1.1 of this trial in April 2019. This non-inferiority trial opened in May 2019 and is expected to be completed in December 2027. The first patient was enrolled in July 2019 and the actual number of enrolled patients was 36 in December 2022. Because of the coronavirus disease 2019 pandemic in Taiwan, the number of enrolled patients in this trial was relatively low. Considering adding the co-investigators and increasing the documents of the participating site, the current protocol has been revised as version 2.0 and was approved by the Research Ethics Committee C of the National Taiwan University Hospital in December 2020**.**

### Supplementary Information

**Additional file 1: Supplementary Table 1.** Manchester scoring system for identification of a pathogenic BRCA1/2 mutation [14, 15].

## Data Availability

Research data are stored in an institutional repository and will be shared upon request to the corresponding author.
