# Supplementary material for: Radiotherapy versus low-dose tamoxifen following breast-conserving surgery for low-risk and estrogen receptor-positive breast ductal carcinoma in situ: an international open-label randomized non-inferiority trial (TBCC-ARO DCIS Trial)
Source: BMC Cancer. 2023 Sep 14;23:865. doi: 10.1186/s12885-023-11291-6 (PMC10500726; doi:10.1186/s12885-023-11291-6)
Supplement: Supplementary file 1 — Additional file 1: Supplementary Table 1. Manchester scoring system for identification of a pathogenic BRCA1/2 mutation [14, 15]. [file 12885_2023_11291_MOESM1_ESM.pdf]

Supplementary Table 1. Manchester scoring system for identification of a pathogenic *BRCA1/2* mutation [Ref.[14,15]]

Cancer, age at diagnosis	*BRCA1*	*BRCA2*
FBC, < 30	6	5
FBC, 30-49	4	4
FBC, 40-49	3	3
FBC, 50-59	2	2
FBC,> 59	1	1
MBC, < 60	5 (if BRCA2 already tested)	8
MBC, > 59	5 (if BRCA2 already tested)	5
Ovarian cancer, < 60	8	5 (if BRCA1 already tested)
Ovarian cancer, > 59	5	5 (if BRCA1 already tested)
Pancreatic cancer	0	1
Prostate cancer, < 60	0	2
Prostate cancer, > 59	0	1

BRCA: breast cancer susceptibility gene.

Family history; FBC, female breast cancer; MBC, male breast cancer
